# TRIQ: a new method to evaluate triclusters

**DOI:** 10.1186/s13040-018-0177-5

**Published:** 2018-08-06

**Authors:** David Gutiérrez-Avilés, Raúl Giráldez, Francisco Javier Gil-Cumbreras, Cristina Rubio-Escudero

**Affiliations:** 1 School of Engineering, Pablo de Olavide University, Seville, Spain; 2 Department of computer Science, University of Seville, Seville, Spain

**Keywords:** Triclustering, Quality measure, Genetic algorithms, Biological quality, Graphical quality, Correlation

## Abstract

**Background:**

Triclustering has shown to be a valuable tool for the analysis of microarray data since its appearance as an improvement of classical clustering and biclustering techniques. The standard for validation of triclustering is based on three different measures: correlation, graphic similarity of the patterns and functional annotations for the genes extracted from the Gene Ontology project (GO).

**Results:**

We propose *TRIQ*, a single evaluation measure that combines the three measures previously described: correlation, graphic validation and functional annotation, providing a single value as result of the validation of a tricluster solution and therefore simplifying the steps inherent to research of comparison and selection of solutions. *TRIQ* has been applied to three datasets already studied and evaluated with single measures based on correlation, graphic similarity and GO terms. Triclusters have been extracted from this three datasets using two different algorithms: TriGen and OPTricluster.

**Conclusions:**

*TRIQ* has successfully provided the same results as a the three single evaluation measures. Furthermore, we have applied *TRIQ* to results from another algorithm, *OPTRicluster*, and we have shown how TRIQ has been a valid tool to compare results from different algorithms in a quantitative straightforward manner. Therefore, it appears as a valid measure to represent and summarize the quality of tricluster solutions. It is also feasible for evaluation of non biological triclusters, due to the parametrization of each component of *TRIQ*.

## Background

Analysis of data structured in 3D manner is becoming an essential task in fields such as biomedical research, for instance in experiments studying gene expression data taking time into account. There is a lot of interest in this type of longitudinal experiments because they allow an in-depth analysis of molecular processes in which the time evolution is important, for example, cell cycles, development at the molecular level or evolution of diseases [1]. Therefore, the use of specific tools for data analysis in which genes are evaluated under certain conditions considering the time factor becomes necessary. In this sense, triclustering [2] appears as a valuable tool since it allows for the assessment of genes under a subset of the conditions of the experiment and under a subset of time points.

The evaluation of solutions obtained by triclustering algorithms is challenging by the fact that there is no ground truth to describe triclusters present in real 3D data. In literature, the standard measures to evaluate tricluster solutions are based on three areas as can be seen in the triclustering publications [3–7]. First, correlation measures such as Pearson [8] or Spearman [9]. Second, graphic validation of the patterns extracted based on the graphic representation, i.e., how similar the genes from a tricluster are based on the graphic representation of the genes across conditions and time points. Third, functional annotations extracted from the Gene Ontology project (GO) [10] for the genes in the tricluster.

However, we consider that providing a single evaluation measure capable of combining the information from the three aforementioned sources of validation is a neccesary task. Therefore, in this work we propose *TRIQ*, a validation measure which combines the three previously proposed validation mechanisms (correlation, graphic validation and functional annotation of the genes).

The application of clustering and biclustering techniques to gene expression data has been broadly studied in the literature [11, 12]. Although triclustering is the result from the natural evolution of the clustering and biclustering techniques, is still a very recent concept. However, nowadays, these techniques are arousing a great interest from the scientific community, which has caused a notable increase of the number of researches focused on finding new triclustering approaches. This section is to provide a general overview of triclustering published in literature. We particularly focus on the validation methods applied to assess the quality of the triclusters obtained.

In 2005, Zhao and Zaki [3] introduced the triCluster algorithm to extract patterns in 3D gene expression data. They presented a measure to assess triclusters’s quality based on the symmetry property. They validated their triclusters based on their graphical representation and Gene Ontology (GO) results. g-triCluster, an extended and generalized version of Zhao and Zaki’s proposal, was published one year later [4]. The authors claimed that the symmetry property is not suitable for all patterns present in biological data and proposed the Spearman rank correlation [9] as a more appropriate tricluster evaluation measure. They also showed validation results based on GO.

An evolutionary computation proposal was made in [13]. The fitness function defined is a multi-objective measure which tries to optimize three conflicting objectives: clusters size, homogeneity and gene-dimension variance of the 3D cluster. The tricluster quality validation was based on GO. LagMiner was introduced in [6] to find time-lagged 3D clusters, what allows to find regulatory relationships among genes. It is based on a novel 3D cluster model called *S*_2_*D*_3_ Cluster. They evaluated their triclusters on homogeneity, regulation, minimum gene number, sample subspace size and time periods length. Their validation was based on graphical representation and GO results. Hu et al. presented an approach focusing on the concept of Low-Variance 3-Cluster [5], which obeys the constraint of a low-variance distribution of cell values. This proposal uses a different functional enrichment tool called CLEAN [14], which uses GO as one of their components. The work in [7] was focused on finding Temporal Dependency Association Rules, which relate patterns of behavior among genes. The rules obtained are used to represent regulated relations among genes. They also validated their triclusters based on their graphical representation and GO results.

Tchagang et al. [15] proposed OPTricluster, a triclustering algorithm which obtains 3D short time series gene expression datasets by applying a statistical methodology. In this case, the authors carried out an in-depth biological validation based on GO, but they tested the robustness of OPTricluster to noise using the Adjusted Rand Index (ARI) [16], which also was used by aforementioned g-tricluster.

In 2013, two new and very interesting approaches were proposed. On the one hand, the *δ*−*TRIMAX* algorithm [17], which applies a variant of the MSR adapted to 3D datasets and yields triclusters that have a MSR score below a threshold *δ*. This algorithm has a version based on evolutionary multi-objective optimization, named *EMOA*−*δ*−*TRIMAX* [18], which aims at optimizing the use of *δ*−*TRIMAX* by adding the capabilities of evolutionary algorithms to retrieve overlapping triclusters. On the other hand, OAC-Triclustering was also proposed by Gnatyshak et al. in [19]. In the following years, the authors developed improvements and extensions of this algorithm [20–22].

More recent works have extended the capabilities of the tricluster algorithms by combination of several approaches. Thereby, Liu et al. [23] mixed fuzzy clustering and fuzzy biclustering algorithms in order to expands them to support 3D data and they used the *F*-Measure and Entropy as criteria to evaluate the performance. Also, Kakati et al. [24] combined parallel biclustering and distributed triclustering approaches to obtain improvements on the computational cost. In this work, the authors use a quality measure based on shifting and scaling patterns [25] to optimize the triclusters obtained.

Most of the methods studied base the quality of the triclusters on the graphic representation or on metrics aimed at measuring diverse characteristics of such representation. From a biological point of view, the standard for validation of triclusters quality is based on GO functional annotations.

## Methods

This section presents the *TRIQ* (TRIcluster Quality) validation measure [26], a novel method to evaluate the quality of triclusters extracted from gene expression datasets.

From an overall perspective, *TRIQ* takes into account the three principal components of a tricluster, i.e. the genes, experimental conditions and time points, in order to measure its quality from three approaches: the level of biological notoriety of the cluster (biological quality), the graphic quality of the patterns of the genes in the tricluster (graphic quality), and the level of correlation of the genes in the tricluster by means of the Pearson [8] and the Spearman [9] indexes. Therefore, *TRIQ* is composed by a combination of four indexes: *BIOQ* (BIOlogical Quality), *GRQ* (GRaphic Quality), *PEQ* (PEarson Quality) and *SPQ* (SPearman Quality).

In Eq. 1 we define *TRIQ* as the weighted sum of each of the four aforementioned terms. Therefore, four associated weights must be defined: the weight for *BIOQ*, denoted as *W*_*bio*_; the weight for *GRQ*, denoted as *W*_*gr*_; the weight for *PEQ*, denoted as *W*_*pe*_; and the weight for *SPQ*, denoted as *W*_*sp*_.

1 $$ \begin{aligned} TRIQ(TRI) &= \frac{1}{W_{bio}+W_{gr}+W_{pe}+W_{sp}} * \left[ W_{bio}*BIOQ(TRI) \right.\\ &\left.+ W_{gr}*GRQ(TRI) + W_{pe}*PEQ(TRI) + W_{sp}*SPQ(TRI)\right] \\ \end{aligned}  $$

**Fig. 1:**
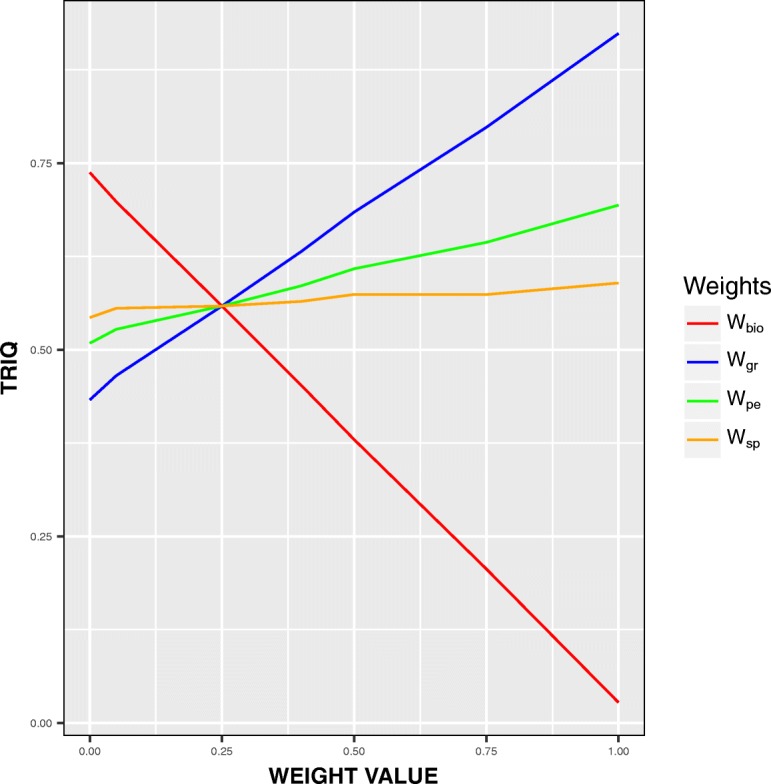
Representation of *BIOQ*, *GRQ*, *PEQ*, and *SPQ* influence on *TRIQ*

This is a general definition of *TRIQ*. In order to obtain a *TRIQ* index as balanced as possible among the four quality indexes *BIOQ*, *GRQ*, *PEQ*, and *SPQ* we performed an exhaustive testing procedure with well known datasets. Several combinations of values of *BIOQ*, *GRQ*, *PEQ*, and *SPQ* were tested, and in Fig. 1 we show the results obtained.

We see that that the value of *TRIQ* is slightly directly dependent on the weights related to correlation, *PEQ*, and *SPQ*. This is due to the fact that these values rank in the [0-1] interval, being usually high, from 0.7 to 1. The value of *TRIQ* has a higher level of dependence to the graphical quality, *GRQ*, and reverse strong dependence to the biological quality, *BIOQ*, due to the fact that *BIOQ* ranks in low values, usually around 10^−3^ to 10^−5^. Based on this experiments, we have configured the *TRIQ* measure with the weights showed in Eq. 2 in order to obtain a balanced value of *TRIQ*.

2 $$ W_{bio} = 0.5, W_{gr} = 0.4, W_{pe} = 0.05, W_{sp} = 0.05  $$

Next, we describe in depth each of the terms involved in the *TRIQ* measure.

### Correlation measures: PEQ and SPQ

The correlation measures involved in *TRIQ* are Pearson’s *PEQ* [8] and Spearman’s *SPQ* [9] correlations. They have been chosen since they are the standard correlation measures and they are widely used in literature [4]. The correlation provides a numerical estimation of the dependence among the genes, conditions and times in the tricluster solutions.

Given a tricluster *TRI*, we compute *PEQ* and *SPQ* by the following mechanism. Given the subset of genes (see Eq. 3a), conditions (see Eq. 3b) and time stamps (see Eq. 3c), we obtain a value of expression for each combination gene, condition and time. For instance, for a tricluster consisting of four genes, two conditions and three time points, we have twenty four expression values. We then compute the Pearson correlation for each pair of values, and compute *PEQ* as the average of the absolute values to avoid negative and positive correlations canceling each other (see Eq. 4). Furthermore, for this measure we do not care if the correlation is positive or negative between values, we only want to know the level of correlation. The *SPQ* value is the equivalent using the Spearman correlation (see Eq. 5).

3a $$\begin{array}{@{}rcl@{}} TRI_{G} &=& <g_{0}, g_{1}, \ldots, g_{G}>\end{array} $$

3b $$\begin{array}{@{}rcl@{}} TRI_{C} &=& <c_{0}, c_{1}, \ldots, c_{C}>\end{array} $$

3c $$\begin{array}{@{}rcl@{}} TRI_{T} &=& <t_{0}, t_{1}, \ldots, t_{T}>\end{array} $$

4 $$ PEQ(TRI) = \frac{\sum_{i=0,j=0}^{\#exp} \left|Pearson_{i \neq j}\left(exp_{i}, exp_{j}\right)\right|}{\#pairs\;of\;exp}  $$

5 $$ SPQ(TRI) = \frac{\sum_{i=0,j=0}^{\#exp} \left|Spearman_{i \neq j}\left(exp_{i}, exp_{j}\right)\right|}{\#pairs\;of\;exp}  $$

with *exp* representing the expressions in each tricluster *TRI*.

### Graphical validation: GRQ

The *GRQ* member of Eq. 1 measures the graphical quality of the tricluster. This graphical quality of a tricluster is a quantitative representation of a qualitative measure: how homogeneous the members of the tricluster are. This method is widely used in literature for visual validation of the results by means of graphically representing the triclusters on their three components: genes, conditions and time points [3, 6, 7].

The *GRQ* index is described in Eq. 6. This measure is defined based on the normalization of the angle value given by *MSL*. The Multi SLope (*MSL*) evaluation function was defined in [27] and, given a tricluster *TRI*, provides a numerical value of the similarity among the angles of the slopes formed by each profile shaped by the genes, conditions, and times of the tricluster.

6 $$ GRQ(TRI) = 1 - \frac{MSL(TRI)}{2\pi}  $$

**Fig. 2:**
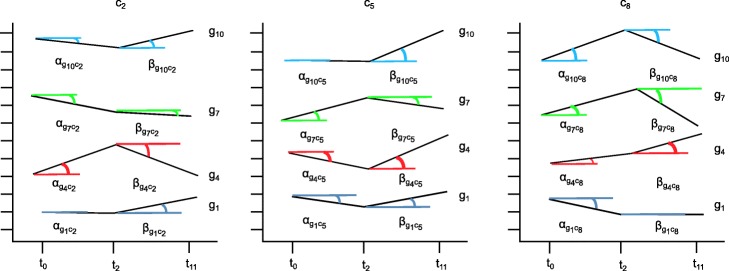
Representation of how the *MSL* measure is calculated. This figure shows three graphics containing four lines each one with a representation of their slopes

The *MSL* measure considers the three graphical views of a tricluster, also defined in [27]: *TRI*_*gct*_, *TRI*_*gtc*_, and *TRI*_*tgc*_. These three terms are generally defined as *TRI*_*xop*_, with the expression levels of the tricluster represented in the Y axis, *x* represents the tricluster component in the X axis (genes or time points), *o* represents the lines plotted in the graph (genes, conditions or time lines) and *p* the type of facets or panels represented (time points or conditions). We can observe an example of the *TRI*_*tgc*_ view of a tricluster with the genes *g*_1_, *g*_4_, *g*_7_ and *g*_10_, the experimental conditions *c*_2_, *c*_5_ and *c*_8_ and the time points *t*_0_, *t*_2_, *t*_11_ in Fig. 2 and see how each line or gene forms a set of angles (two for this particular example) defined by each time point in the X axis for every panel or experimental condition. Thus, *MSL* measures the differences among the angles formed by every series traced on each of the three graphic representations taking into account *TRI*_*gct*_, *TRI*_*gtc*_, and *TRI*_*tgc*_. A near to zero value of *MSL* implies a better graphical quality of a tricluster therefore, according to *GRQ* formulation in Eq. 6, a tricluster is graphically better the smaller the value of *MSL*.

### Biological validation: BIOQ

The *BIOQ* member of Eq. 1 measures the biological quality of the tricluster. Specifically, *BIOQ* uses the genes (*TRI*_*G*_) of the input tricluster *TRI* to compute this index. As you can see in Eq. 7, the biological quality of a tricluster *TRI* is defined as the biological significance, *SIG*_*bio*_, of the set of genes *TRI*_*G*_ divided by the *S*_*max*_ value.

7 $$ BIOQ(TRI) = \frac{SIG_{bio}\left(TRI_{G}\right)}{S_{max}}  $$

The *SIG*_*bio*_ and *S*_*max*_ elements of the *BIOQ* index have been designed in order to represent, by means of a quantitative score, the value of the Gene Ontology analysis of the genes that compose the measured tricluster.

The Gene Ontology Project (GO) [10] is a major bioinformatics initiative with the aim of standardizing the representation of gene and gene product attributes across species and databases, besides identifying the annotated terms, performs the statistical analysis for the over-representation of those terms, also providing a statistical significance *p*-value. However, it is also important to take into account how deep in the ontology the terms are annotated, with the deeper terms being more specific than the superficial ones [28]. The *SIG*_*bio*_ and *S*_*max*_ elements are calculated based on the GO analysis that identifies, for a set of genes in a tricluster, the terms listed in each of the three available ontologies: biological processes, cellular components, and molecular functions. This GO analysis is performed with the software Ontologizer [29].

The computation of *SIG*_*bio*_ consists on counting how many terms of the annotated genes of the tricluster in the GO analysis are in a particular intervals of *p*-value. Table 1 represents the ah-hoc designed system of intervals of *p*-value and scoring system. The intervals and the scoring system are defined in Eq. 8 where for a given level, *Inter*_*l*_ is defined by a weight value *w*_*l*_ for the level, and by the lower and upper bounds (*inf*_*l*_ and *sup*_*l*_, respectively), being an open-closed *p*-values interval (Eq. 8a). The set of existing *LV* consists of all levels with *Inf*_*l*_ smaller or equal to a minimum *p*-value, *th*. For each interval of each level *Inter*_*l*_, the weight value *w*_*l*_ is defined in Eq. 8c; *Inf*_*l*_ is defined in Eq. 8d, and *sup*_*l*_ is defined in Eq. 8e.

**Table 1:** Biological significance intervals

Level (l)	Weight (*w*_*l*_)	Interval (*inter*_*l*_)
41	401	(0.0E-00,1.0E-40]
40	391	(1.0E-40,1.0E-39]
39	381	(1.0E-39,1.0E-38]
38	371	(1.0E-38,1.0E-37]
37	361	(1.0E-37,1.0E-36]
36	351	(1.0E-36,1.0E-35]
35	341	(1.0E-35,1.0E-34]
34	331	(1.0E-34,1.0E-33]
33	321	(1.0E-33,1.0E-32]
32	311	(1.0E-32,1.0E-31]
31	301	(1.0E-31,1.0E-30]
30	291	(1.0E-30,1.0E-29]
29	281	(1.0E-29,1.0E-28]
28	271	(1.0E-28,1.0E-27]
27	261	(1.0E-27,1.0E-26]
26	251	(1.0E-26,1.0E-25]
25	241	(1.0E-25,1.0E-24]
24	231	(1.0E-24,1.0E-23]
23	221	(1.0E-23,1.0E-22]
22	211	(1.0E-22,1.0E-21]
21	201	(1.0E-21,1.0E-20]
20	191	(1.0E-20,1.0E-19]
19	181	(1.0E-19,1.0E-18]
18	171	(1.0E-18,1.0E-17]
17	161	(1.0E-17,1.0E-16]
16	151	(1.0E-16,1.0E-15]
15	141	(1.0E-15,1.0E-14]
14	131	(1.0E-14,1.0E-13]
13	121	(1.0E-13,1.0E-12]
12	111	(1.0E-12,1.0E-11]
11	101	(1.0E-11,1.0E-10]
10	91	(1.0E-10,1.0E-09]
9	81	(1.0E-09,1.0E-08]
8	71	(1.0E-08,1.0E-07]
7	61	(1.0E-07,1.0E-06]
6	51	(1.0E-06,1.0E-05]
5	41	(1.0E-05,1.0E-04]
4	31	(1.0E-04,1.0E-03]
3	21	(1.0E-03,1.0E-02]
2	11	(1.0E-02,1.0E-01]
1	1	(1.0E-01,1.0E-00]


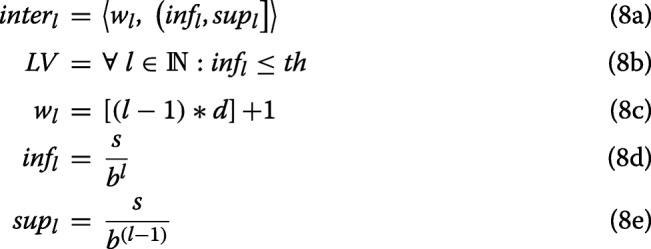


This definition is made in order to establish a general interval system dependent on the parameters described above. For our purpose, we have settled these parameters as shown in Eq. 9; this configuration produces the intervals of Table 1, furthermore, it describes all the biological significance intervals for the configuration detailed in Eq. 9. For each row, weight (*wl*) and range (*inter*_*l*_) for each level (*L*) sorted in ascending order are shown. Each interval provides a set of *p*-values where their significance is directly related to the corresponding level, that is, a *p*-value is better the higher the level to which it belongs, and a *p*-value is better the closer to zero it is.

9a $$\begin{array}{@{}rcl@{}} th &=& 1.0\times 10^{-40} \end{array} $$

9b $$\begin{array}{@{}rcl@{}} d &=& 10.0 \end{array} $$

9c $$\begin{array}{@{}rcl@{}} b &=& 10.0 \end{array} $$

9d $$\begin{array}{@{}rcl@{}} s &=& 1.0 \end{array} $$

9e $$\begin{array}{@{}rcl@{}} LV &=& \lbrace 1,\ldots,41 \rbrace \end{array} $$

Taking into account each level *l* and each predefined interval *inter*_*l*_, the biological significance for the genes of the measured tricluster is defined in Eq. 10a as the addition of all scores for each level *l* from the *LV* level set Eq. 9e. The score function *S* for a level *l* (Eq. 10b) is defined by the multiplication of the concentration of terms for this level *C*(*l*), defined in Eq. 10c as the number of terms of the level *l* divided by the total number of terms, by the weight of the level, and by the level plus a bonus function *f*_*bonus*_, defined in Eq. 10d as the sum of the level plus a bonus value *V*_*bonus*_ if the current level is the maximum level of *LV* or zero in any another case.

10a $$\begin{array}{@{}rcl@{}} SIG_{bio} (TRI_{G}) &=& \sum_{l \in LV} S(l) \end{array} $$

10b $$\begin{array}{@{}rcl@{}} S(l) &=& C(l)*w_{l}*l + f_{bonus}(l) \end{array} $$

10c $$\begin{array}{@{}rcl@{}} C(l) &=& \frac{\#terms(l)}{\#total\;terms} \end{array} $$

10d $$\begin{array}{@{}rcl@{}} f_{bonus}(l) &=& if\; \left(l \;equal\; to\; l_{max}\right)\; then\;\; l + V_{bonus}\;\; else 0 \end{array} $$

Again, this definition is made in order to establish a general system of *SIG*_*bio*_. For our purpose and as a result of an exhaustive testing, the *V*_*bonus*_ parameter has been settled to 0; this fact produces *S*_*max*_ as the maximum achievable score for the interval configuration as you can see in Eq. (11), that has been used to the *SIG*_*bio*_ normalization in Eq. 7.

11 $$ S_{max} = \left(C_{max} * w_{l_{41}} * l_{41}\right) + f_{bonus}(41) = (1 * 401 * 41) + (41 + 0) = 16482  $$

## Results

In this section, we present how *TRIQ* works in an experimental environment. To reach this goal, we have used the TriGen algorithm [2] and the OPTricluster algorithm [15] in order to analyze the datasets, find triclusters and measure them with *TRIQ*.

*TriGen* is based on an heuristic, genetic algorithm, and its performance greatly depends on the fitness function used to find the triclusters. There are three fitness functions available in *TriGen*: Mean Squared Residue 3D (*MSR*_3*D*_) [30], Least Squared Lines (*LSL*) [31] and Multi SLope Measure (*MSL*) [27]. *OPTricluster* identifies triclusters of genes with expression levels having the same direction across the time point experiments in subsets of samples taking into consideration the sequential nature of the time-series.

The three datasets analyzed that involve genes and experimental conditions examined under certain time points are:

- $D_{elu_{3D}}$: The yeast cell cycle *(Saccharomyces Cerevisiae)* [32], in particular, the elutriation experiment.
- $D_{GDS4510_{3D}}$: The GDS4510 dataset from an experiment with mice (Mus Musculus) [33].
- $D_{GSD4472_{3D}}$: The GDS4472 dataset from an experiments with humans (Homo Sapiens) [34].

The first dataset is available at the Stanford University website. The last two datasets have been retrieved from Gene Expression Omnibus [35], a repository of high throughput gene expression data.

For each dataset, we have performed four algorithm executions: *TriGen* with *MSR*_3*D*_ (hereon *MSR*_3*D*_), *TriGen* with *LSL* (hereon *LSL*), *TriGen* with *MSL* and (hereon *MSL*) and *OPTricluster* (hereon *OPT*).

For each algorithm execution and dataset, we have yielded 10 triclusters and the *TRIQ* measure has been used to evaluate their quality. We have found 10 triclusters for each execution in order to have a high number of solutions where *TRIQ* can show its suitability. In the case of *MSR*_3*D*_, *LSL*, and *MSL* executions the number of triclusters has been chosen as one of the *TriGen* algorithm parameters and for *OPT* executions, the tricluster have been randomly selected from the wide collection of triclusters yielded.

Summarizing, we present three experimental batches (*Yeast Elutriation Dataset*, *Mouse GDS4510 Dataset* and, *Human GDS4472 Dataset*) with four experiments each one: *MSR*_3*D*_, *LSL*, *MSL* and *OPT*.

### Yeast elutriation dataset

This batch corresponds to the yeast *(Saccharomyces Cerevisiae)* cell cycle problem [32]. The yeast cell cycle analysis project’s goal is to identify all genes whose mRNA levels are regulated by the cell cycle. The resources used are public and available in http://genome-www.stanford.edu/cellcycle/. There, we can find information relative to gene expression values obtained from different experiments using microarrays.

For our purpose, we have created a dataset $D_{elu_{3D}}$ from the elutriation experiment with 7744 genes, 13 experimental conditions, and 14 time points. Experimental conditions correspond to different statistical measures of the Cy3 and Cy5 channels while time points represent different moments of taking measures from 0 to 390 min.

$D_{elu_{3D}}$ has been used as the input of the *TriGen* and the *OPTtricluster* algorithm in four experiments: *MSR*_3*D*_, *LSL*, *MSL* and, *OPT*.

#### Elutriation *MSR*_3*D*_ experiment

We can verify in Table 2 how *TRI*_9_ has the best values of *BIOQ*, *PEQ* and *SPQ* whereas *TRI*_10_ has the best value of *GRQ*. The *GRQ*, *PEQ* and *SPQ* values are stabilized from *TRI*_2_ to *TRI*_8_ until *TRI*_9_−*TRI*_10_ when these values reach the maximum. Regarding *BIOQ* values, these vary around 0.0012. Furthermore, *TRIQ* values are stable for all solutions except *TRI*_9_−*TRI*_10_ due to the genetic algorithms nature. In conclusion, *TRI*_9_ is the best solution since it has the best value of *TRIQ*, closely followed by *TRI*_10_.

**Table 2:** *MSR*_3*D*_ Elutriation solution table

SOLUTION	TRIQ	BIOQ	GRQ	PEQ	SPQ
*T* *R* *I*_1_	0.289957861	0.001180518	0.627911696	0.400860192	0.363198285
*T* *R* *I*_2_	0.283154367	0.001118227	0.610190268	0.397890126	0.372492792
*T* *R* *I*_3_	0.292658244	0.001217404	0.632360796	0.39778358	0.384320901
*T* *R* *I*_4_	0.283891286	0.001085482	0.614807027	0.38713593	0.36137875
*T* *R* *I*_5_	0.282839356	0.001224203	0.613862367	0.379462124	0.354184014
*T* *R* *I*_6_	0.290639052	0.001129778	0.625267377	0.4293412	0.370003051
*T* *R* *I*_7_	0.259777538	0.001208157	0.553613841	0.382072259	0.372486191
*T* *R* *I*_8_	0.281909708	0.001215203	0.606726347	0.407953984	0.36427737
*T* *R* *I*_9_	0.453932884	0.001330144	0.896650615	0.986952953	0.905198358
*T* *R* *I*_10_	0.45152166	0.001148045	0.934659815	0.776480244	0.765193987

#### Elutriation *LSL* experiment

In Table 3 you can see how *TRI*_3_ has the best value of *BIOQ*, *TRI*_2_ has the best value of *GRQ*, *TRI*_6_ has the best value of *PEQ* and, *TRI*_1_ has the best value of *SPQ*. In general, the *GRQ*, *PEQ* and *SPQ* values vary around an average value from *TRI*_1_ until *TRI*_8_. Then, these values decrease in *TRI*_9_−*TRI*_10_ solutions due to the fact that the algorithm reached a local minimum in this two solutions; the *BIOQ* values fluctuate around 0.0012 value reaching a maximum in *TRI*_3_ and a minimum in *TRI*_4_. The values of *TRIQ* reach the maximum values at the first two solutions, then remain stable and finally fall in local minimum in the last two solutions. In conclusion, *TRI*_1_ is the best solution since it has the best value of *TRIQ*.

**Table 3:** *LSL* Elutriation solution table

SOLUTION	TRIQ	BIOQ	GRQ	PEQ	SPQ
*T* *R* *I*_1_	0.444841672	0.001147115	0.925741144	0.737449684	0.741983455
*T* *R* *I*_2_	0.444050729	0.001217804	0.927628308	0.725178526	0.722631553
*T* *R* *I*_3_	0.434940552	0.001327826	0.912385527	0.697309668	0.689138899
*T* *R* *I*_4_	0.431591352	0.001071675	0.905144571	0.692097513	0.6878562
*T* *R* *I*_5_	0.433960732	0.001125858	0.913689264	0.683063155	0.675378795
*T* *R* *I*_6_	0.440497687	0.001192667	0.916680329	0.743684691	0.720899755
*T* *R* *I*_7_	0.437721769	0.001143537	0.916956452	0.702066665	0.705281726
*T* *R* *I*_8_	0.441054484	0.001233014	0.919127603	0.730818495	0.724920229
*T* *R* *I*_9_	0.41970611	0.001200657	0.894690897	0.629273489	0.595314967
*T* *R* *I*_10_	0.399331119	0.001102605	0.848695823	0.597009139	0.589020606

#### Elutriation *MSL* experiment

We can observe in Table 4 how *TRI*_2_ has the best value of *BIOQ*, *PEQ* and *SPQ* whereas *TRI*_1_ has the best value of *GRQ*. The *GRQ*, *PEQ* and *SPQ* have a stable fluctuation throughout the solutions whilst *BIOQ* varying around the central value 0.0011. The *TRIQ* values reach their maximum value at *TRI*_2_, the minimum at *TRI*_3_ and the rest are stabilized. In conclusion, *TRI*_2_ is the best solution since it has the best value of *TRIQ*.

**Table 4:** *MSL* Elutriation solution table

SOLUTION	TRIQ	BIOQ	GRQ	PEQ	SPQ
*T* *R* *I*_1_	0.492819589	0.001051563	0.999519361	0.929642164	0.9200791
*T* *R* *I*_2_	0.493539244	0.001240807	0.997800501	0.930758605	0.945214193
*T* *R* *I*_3_	0.476117422	0.001118134	0.991760508	0.78527775	0.791805282
*T* *R* *I*_4_	0.478990452	0.001166044	0.991627468	0.813400974	0.821727882
*T* *R* *I*_5_	0.480938627	0.001090473	0.995151057	0.820019348	0.826640002
*T* *R* *I*_6_	0.475974935	0.001085123	0.992527638	0.779644523	0.788781847
*T* *R* *I*_7_	0.478754345	0.00100258	0.994551592	0.806892319	0.801756043
*T* *R* *I*_8_	0.478200414	0.001199622	0.993176848	0.804634565	0.801962727
*T* *R* *I*_9_	0.477639873	0.001147773	0.991707562	0.805881226	0.801778004
*T* *R* *I*_10_	0.475505918	0.001132268	0.989937077	0.788265502	0.791033557

#### Elutriation *OPT* experiment

We can verify in Table 5 how all triclusters have the same value of *BIOQ* since all triclusters grouped the same collection of genes. Regarding *GRQ* index, the triclusters have values between 0.70 and 0.86 with the exception of *TRI*_1_, *TRI*_9_ and, *TRI*_8_ being *TRI*_4_ the solution with better *GRQ*. The *PEQ* and *SPQ* indexes have fluctuating values being *TRI*_7_ the tricluster with the better *PEQ* and *SPQ*. In conclusion, *TRI*_7_ is the best solution since it has the best value of *TRIQ*.

**Table 5:** *OPT* Elutriation solution table

SOLUTION	TRIQ	BIOQ	GRQ	PEQ	SPQ
*T* *R* *I*_1_	0.25439082	0.000728	0.55556687	0.32575013	0.31025512
*T* *R* *I*_2_	0.31786223	0.000728	0.7005279	0.36494172	0.38080349
*T* *R* *I*_3_	0.38238284	0.000728	0.84763736	0.40215787	0.45712372
*T* *R* *I*_4_	0.39914764	0.000728	0.86882797	0.49884203	0.52621082
*T* *R* *I*_5_	0.39749144	0.000728	0.86565058	0.50040614	0.51694181
*T* *R* *I*_6_	0.40017866	0.000728	0.86455717	0.53452807	0.5453116
*T* *R* *I*_7_	0.40707391	0.000728	0.84656685	0.66128956	0.70037758
*T* *R* *I*_8_	0.25896921	0.000728	0.56897207	0.31626703	0.30406432
*T* *R* *I*_9_	0.25904229	0.000728	0.56900655	0.31749708	0.30402
*T* *R* *I*_10_	0.32249076	0.000728	0.72222718	0.33095502	0.33376653

#### Elutriation summary

**Fig. 3:**
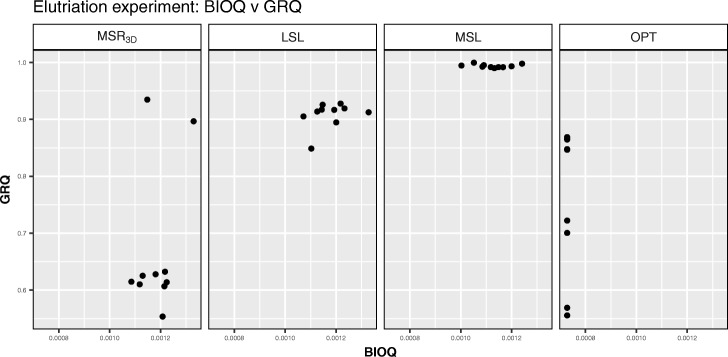
*BIOQ* vs *GRQ* dispersion graph for each Elutriation solution of each experiment

**Fig. 4:**
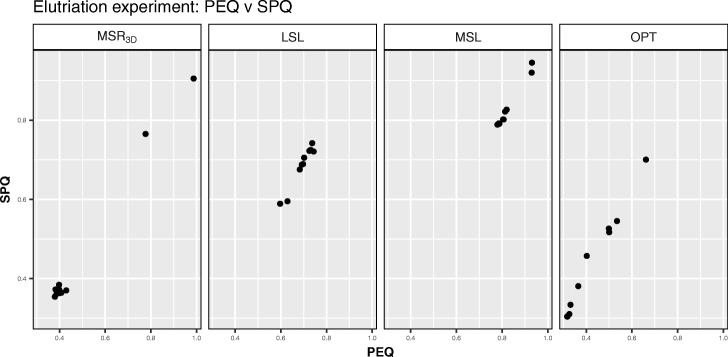
*PEQ* vs *SPQ* dispersion graph for each Elutriation solution of each experiment

We can see in Fig. 3 how the solutions are distributed regarding *BIOQ* and *GRQ* for each experiment. We observe that all points are concentrated in a *BIOQ* interval of [0.000728,0.0013] for each experiment meanwhile the *MSL* experiment stands out because all its solutions have a *GRQ* near to 1. Regarding the *PEQ* and *SPQ* solutions distribution, we can see in Fig. 4 how the majority of the solutions are concentrated around the point *PEQ*=0.325,*SPQ*=0.325 in the *MSR*_3*D*_ experiment, all solutions are concentrated in [0.50,0.75] interval for *PEQ* and *SPQ* in the *LSL* experiment, all solutions are concentrated in [0.75,1.00] interval for *PEQ* and *SPQ* in the *MSL* experiment and, all solutions are concentrated in [0.30,0.70] interval for *PEQ* and *SPQ* in the *OPT* experiment.

The global *TRIQ*-based ranking of solutions is showed in Table 6; we can see how the solutions of the *MSL* experiment are placed on the first positions followed by two outstanding solutions of the *MSR*_3*D*_ experiment, all solutions of the *LSL* experiment, all solutions of the *OPT* experiment and, the remaining solutions of the *MSR*_3*D*_ experiment.

**Table 6:** Elutriation ranking table

EXPERIMENT	SOLUTION	TRIQ	BIOQ	GRQ	PEQ	SPQ
*MSL*	*T* *R* *I*_2_	0.493539244	0.001240807	0.997800501	0.930758605	0.945214193
*MSL*	*T* *R* *I*_1_	0.492819589	0.001051563	0.999519361	0.929642164	0.9200791
*MSL*	*T* *R* *I*_5_	0.480938627	0.001090473	0.995151057	0.820019348	0.826640002
*MSL*	*T* *R* *I*_4_	0.478990452	0.001166044	0.991627468	0.813400974	0.821727882
*MSL*	*T* *R* *I*_7_	0.478754345	0.00100258	0.994551592	0.806892319	0.801756043
*MSL*	*T* *R* *I*_8_	0.478200414	0.001199622	0.993176848	0.804634565	0.801962727
*MSL*	*T* *R* *I*_9_	0.477639873	0.001147773	0.991707562	0.805881226	0.801778004
*MSL*	*T* *R* *I*_3_	0.476117422	0.001118134	0.991760508	0.78527775	0.791805282
*MSL*	*T* *R* *I*_6_	0.475974935	0.001085123	0.992527638	0.779644523	0.788781847
*MSL*	*T* *R* *I*_10_	0.475505918	0.001132268	0.989937077	0.788265502	0.791033557
*M* *S* *R*_3 *D*_	*T* *R* *I*_9_	0.453932884	0.001330144	0.896650615	0.986952953	0.905198358
*M* *S* *R*_3 *D*_	*T* *R* *I*_10_	0.45152166	0.001148045	0.934659815	0.776480244	0.765193987
*LSL*	*T* *R* *I*_1_	0.444841672	0.001147115	0.925741144	0.737449684	0.741983455
*LSL*	*T* *R* *I*_2_	0.444050729	0.001217804	0.927628308	0.725178526	0.722631553
*LSL*	*T* *R* *I*_8_	0.441054484	0.001233014	0.919127603	0.730818495	0.724920229
*LSL*	*T* *R* *I*_6_	0.440497687	0.001192667	0.916680329	0.743684691	0.720899755
*LSL*	*T* *R* *I*_7_	0.437721769	0.001143537	0.916956452	0.702066665	0.705281726
*LSL*	*T* *R* *I*_3_	0.434940552	0.001327826	0.912385527	0.697309668	0.689138899
*LSL*	*T* *R* *I*_5_	0.433960732	0.001125858	0.913689264	0.683063155	0.675378795
*LSL*	*T* *R* *I*_4_	0.431591352	0.001071675	0.905144571	0.692097513	0.6878562
*LSL*	*T* *R* *I*_9_	0.41970611	0.001200657	0.894690897	0.629273489	0.595314967
*OPT*	*T* *R* *I*_7_	0.40707391	0.000728	0.84656685	0.66128956	0.70037758
*OPT*	*T* *R* *I*_6_	0.40017866	0.000728	0.86455717	0.53452807	0.5453116
*LSL*	*T* *R* *I*_10_	0.399331119	0.001102605	0.848695823	0.597009139	0.589020606
*OPT*	*T* *R* *I*_4_	0.39914764	0.000728	0.86882797	0.49884203	0.52621082
*OPT*	*T* *R* *I*_5_	0.39749144	0.000728	0.86565058	0.50040614	0.51694181
*OPT*	*T* *R* *I*_3_	0.38238284	0.000728	0.84763736	0.40215787	0.45712372
*OPT*	*T* *R* *I*_10_	0.32249076	0.000728	0.72222718	0.33095502	0.33376653
*OPT*	*T* *R* *I*_2_	0.31786223	0.000728	0.7005279	0.36494172	0.38080349
*M* *S* *R*_3 *D*_	*T* *R* *I*_3_	0.292658244	0.001217404	0.632360796	0.39778358	0.384320901
*M* *S* *R*_3 *D*_	*T* *R* *I*_6_	0.290639052	0.001129778	0.625267377	0.4293412	0.370003051
*M* *S* *R*_3 *D*_	*T* *R* *I*_1_	0.289957861	0.001180518	0.627911696	0.400860192	0.363198285
*M* *S* *R*_3 *D*_	*T* *R* *I*_4_	0.283891286	0.001085482	0.614807027	0.38713593	0.36137875
*M* *S* *R*_3 *D*_	*T* *R* *I*_2_	0.283154367	0.001118227	0.610190268	0.397890126	0.372492792
*M* *S* *R*_3 *D*_	*T* *R* *I*_5_	0.282839356	0.001224203	0.613862367	0.379462124	0.354184014
*M* *S* *R*_3 *D*_	*T* *R* *I*_8_	0.281909708	0.001215203	0.606726347	0.407953984	0.36427737
*M* *S* *R*_3 *D*_	*T* *R* *I*_7_	0.259777538	0.001208157	0.553613841	0.382072259	0.372486191
*OPT*	*T* *R* *I*_9_	0.25904229	0.000728	0.56900655	0.31749708	0.30402
*OPT*	*T* *R* *I*_8_	0.25896921	0.000728	0.56897207	0.31626703	0.30406432
*OPT*	*T* *R* *I*_1_	0.25439082	0.000728	0.55556687	0.32575013	0.31025512

**Fig. 5:**
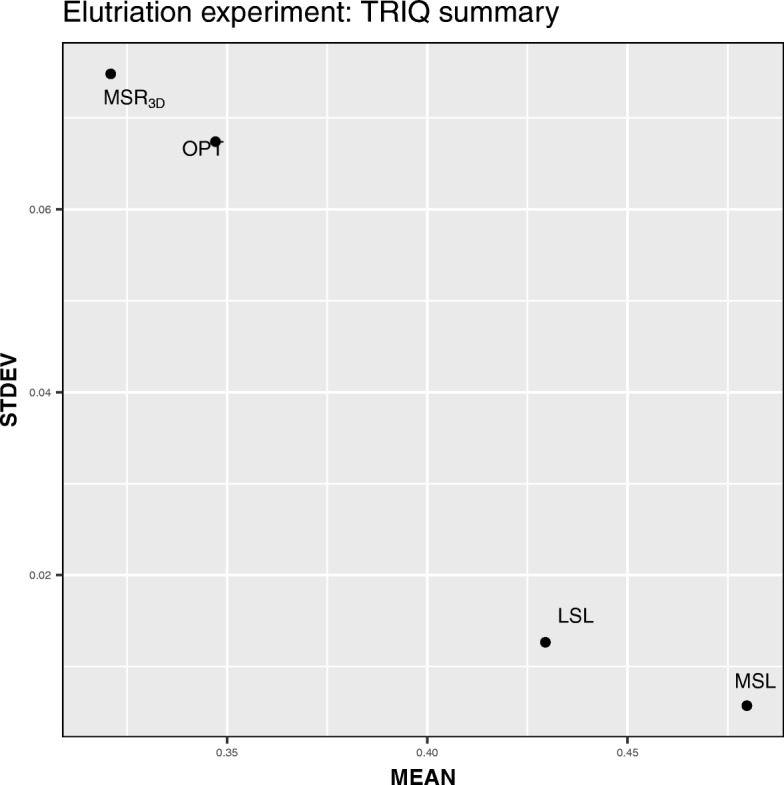
*MEAN* vs *STDEV* dispersion graph for each Elutriation experiment

The *MSL* experiment has the best average values of *TRIQ* and the lowest standard deviation of *TRIQ* as seen in Table 7. This fact is reflected in Fig. 5 wherein the *MSL* point is located on the bottom-right side of the graph which implies that the *MSL* experiment has the highest values of *TRIQ* and a sparsely dispersed distribution, thus this is a high-quality experiment.

**Table 7:** Elutriation summary table

EXPERIMENT	BEST SOLUTION	BEST TRIQ	MEAN	STDEV
*M* *S* *R*_3 *D*_	*T* *R* *I*_9_	0.453932884	0.317028196	0.072095449
*LSL*	*T* *R* *I*_1_	0.444841672	0.432769621	0.013833102
*MSL*	*T* *R* *I*_2_	0.493539244	0.480848082	0.006701521
*OPT*	*T* *R* *I*_7_	0.40707391	0.33990298	0.064949576

**Fig. 6:**
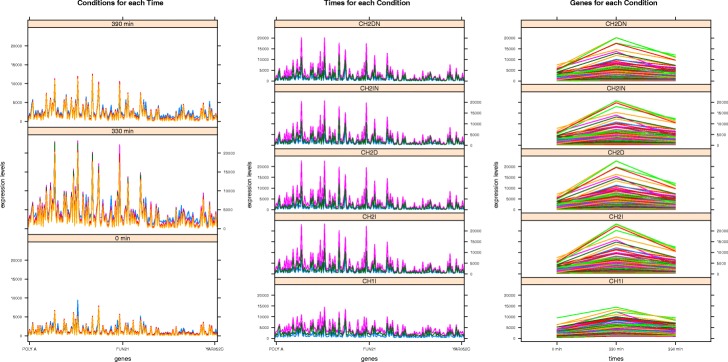
*TRI*_2_ graphic views of the Elutriation *MSL* experiment

The most valuable solution of all experiments is the tricluster *TRI*_2_ of the *MSL* experiment. We can see in Fig. 6 its three graphic views showing that its high value of *GRQ* is reflected in the patterns depicted. Furthermore, in Table 8 we observe terms with moderately low *p*-value as *fermentation*, *vesicle fusion to plasma membrane* and *exocytosis*. *Fermentation* is a biological process that is part of the process called *energy derivation by oxidation of organic compounds* and, in turn, belongs to *the generation of precursor metabolites and energy* process and *the oxidation-reduction process*; *Vesicle fusion to plasma membrane* is a biological process that is part of the *exocytosis proccess*; the first term is a process of *cellular component organization* whereas the second is an *establishment of localization process*.

**Table 8:** *TRI*_2_ GO table of the *MSL* Elutriation experiment

TERM ID	TERM	*P*-VALUE
GO:0006113	Fermentation	7.39E-04
GO:0099500	Vesicle fusion to plasma membrane	0.001183063
GO:0006887	Exocytosis	0.001183063
GO:0140029	Exocytic process	0.001183063
GO:0045026	Plasma membrane fusion	0.00141327
GO:0000145	Exocyst	0.001794132
GO:0048193	Golgi vesicle transport	0.002271213
GO:0061025	Membrane fusion	0.002444417
GO:0051039	Positive regulation of transcription involved in meiotic cell cycle	0.002483587
GO:0051436	Negative regulation of ubiquitin-protein ligase activity involved in mitotic cell cycle	0.002483587
GO:0051439	Regulation of ubiquitin-protein ligase activity involved in mitotic cell cycle	0.002483587
GO:1904667	Negative regulation of ubiquitin protein ligase activity	0.002483587
GO:0032940	Secretion by cell	0.00251212
GO:0046903	Secretion	0.00251212
GO:0051049	Regulation of transport	0.002574368
GO:0061024	Membrane organization	0.002785422
GO:0051321	Meiotic cell cycle	0.003307558
GO:1903046	Meiotic cell cycle process	0.003307558
GO:0140013	Meiotic nuclear division	0.003307558
GO:0044275	Cellular carbohydrate catabolic process	0.004058262

### Mouse GDS4510 dataset

This batch corresponds to the mouse GDS4510 dataset. This dataset was obtained from GEO [35] with accession code GDS4510 and title *rd1 model of retinal degeneration: time course* [33]. In this experiment, the degeneration of retinal cells in different individuals of home mouse *(Mus musculus)* is analyzed over 4 days just after birth, specifically on days 2, 4, 6 and 8.

For our purpose, we have created a dataset $D_{GDS4510_{3D}}$ composed of 22690 genes, 8 experimental conditions (one for each individual involved in the biological experiment) and 4 time points.

$D_{GDS4510_{3D}}$ has been used as the input of the *TriGen* and the *OPTtricluster* algorithm in four experiments: *MSR*_3*D*_, *LSL*, *MSL* and, *OPT*.

#### GDS4510 *MSR*_3*D*_ experiment

We can verify in Table 9 how *TRI*_7_ has the best value of *BIOQ*, *GRQ*, *PEQ*, *SPQ*. The *GRQ*, *PEQ* and *SPQ* indexes vary uniformly among all the solutions. *BIOQ* has a peak of *TRI*_7_ which has the maximum value. The *TRIQ* values oscillate between 0.385 and 0.4 with the exception of *TRI*_7_, therefore this is the best solution since it has the best value of *TRIQ*.

**Table 9:** *MSR*_3*D*_ GDS4510 solution table

SOLUTION	TRIQ	BIOQ	GRQ	PEQ	SPQ
*T* *R* *I*_1_	0.399937853	0.001348086	0.870211819	0.516112583	0.507469069
*T* *R* *I*_2_	0.397972383	0.001177971	0.866535835	0.511042941	0.504338338
*T* *R* *I*_3_	0.391066411	0.001255371	0.849235889	0.508613518	0.506273874
*T* *R* *I*_4_	0.397028323	0.0014405	0.863853884	0.512208068	0.503122322
*T* *R* *I*_5_	0.388644055	0.001187588	0.842929885	0.511734835	0.505831309
*T* *R* *I*_6_	0.392316477	0.00190466	0.850869722	0.513791033	0.506534134
*T* *R* *I*_7_	0.40677296	0.004479209	0.882477468	0.520330064	0.510517317
*T* *R* *I*_8_	0.3851186	0.001240227	0.834606686	0.508323861	0.504792392
*T* *R* *I*_9_	0.390891083	0.001281294	0.848296937	0.510926324	0.507706903
*T* *R* *I*_10_	0.390730352	0.001137925	0.8484396	0.50930819	0.506402803

#### GDS4510 *LSL* experiment

In Table 10 we can see how *TRI*_1_ has the best value of *BIOQ* and *GRQ* meanwhile *TRI*_2_ has the best values of *PEQ* and *SPQ*. The *GRQ*, *PEQ* and *SPQ* values vary uniformly around a central value among the triclusters whereas *BIOQ* has peak values in *TRI*_1_ and *TRI*_4_. The *TRIQ* values oscillates between 0.40 and 0.43 being *TRI*_1_, *TRI*_4_ and *TRI*_9_ the most outstanding solutions. We can conclude that *TRI*_1_ is the best solution since it has the best value of *TRIQ*.

**Table 10:** *LSL* GDS4510 solution table

SOLUTION	TRIQ	BIOQ	GRQ	PEQ	SPQ
*T* *R* *I*_1_	0.435171938	0.005902591	0.902662935	0.6949723	0.728137064
*T* *R* *I*_2_	0.427168871	0.002676716	0.885320349	0.700788434	0.733259027
*T* *R* *I*_3_	0.422560787	0.002909652	0.887599773	0.648196371	0.673124663
*T* *R* *I*_4_	0.42987221	0.004218243	0.901813346	0.657798589	0.68295641
*T* *R* *I*_5_	0.416008121	0.002006917	0.869706286	0.658784335	0.683658641
*T* *R* *I*_6_	0.41490654	0.001678815	0.866767983	0.661174595	0.686024185
*T* *R* *I*_7_	0.417068507	0.001262531	0.87574877	0.648950843	0.673803828
*T* *R* *I*_8_	0.40861261	0.001179271	0.854772399	0.649739272	0.672541022
*T* *R* *I*_9_	0.42454573	0.001683951	0.890635412	0.663209255	0.68578253
*T* *R* *I*_10_	0.417718487	0.002071672	0.874308872	0.657210052	0.681971994

#### GDS4510 *MSL* experiment

For this experiment, we can observe in Table 11 how *TRI*_1_ has the best value of *BIOQ* and *GRQ* meanwhile *TRI*_2_ has the best value of *PEQ* and *TRI*_8_ has the best value of *SPQ*. The *PEQ* and *SPQ* indexes of all solutions vary uniformly around 0.5 whereas all the *GRQ* values are close to 0.9. The *BIOQ* values oscillate between 0.0012 and 0.0019 reaching its higher value in the *TRI*_1_ solution. The *TRIQ* values are in the [0.42,0.44] interval, therefore we can conclude that they are good results for this experiment. The highest value of *TRIQ* is reached by *TRI*_1_, hence it is the best solution for this experiment.

**Table 11:** *MSL* GDS4510 solution table

SOLUTION	TRIQ	BIOQ	GRQ	PEQ	SPQ
*T* *R* *I*_1_	0.446289279	0.003624207	0.990551544	0.496833632	0.468297522
*T* *R* *I*_2_	0.430638622	0.001399471	0.945717127	0.515568434	0.51747227
*T* *R* *I*_3_	0.429698209	0.00149303	0.943951098	0.506740977	0.520684131
*T* *R* *I*_4_	0.425844616	0.001388422	0.935696236	0.506485147	0.510953062
*T* *R* *I*_5_	0.431185402	0.001224915	0.948344121	0.507510722	0.517195194
*T* *R* *I*_6_	0.422692807	0.001367523	0.927464145	0.507112049	0.513355693
*T* *R* *I*_7_	0.429129078	0.001401202	0.944156645	0.501640545	0.513675839
*T* *R* *I*_8_	0.436192976	0.001999141	0.958438573	0.512285251	0.524074273
*T* *R* *I*_9_	0.433173322	0.001604555	0.95182792	0.510885656	0.521911883
*T* *R* *I*_10_	0.422409162	0.001390319	0.928018397	0.501351072	0.508781791

#### GDS4510 *OPT* experiment

In Table 12 we can see how *TRI*_2_ has the best value of *BIOQ*, *TRI*_4_ has the best value of *GRQ* and, *TRI*_9_ and *TRI*_1_ have the best value of *PEQ* and *SPQ* respectively. The *BIOQ* values vary among [0.0012,0.0016] interval with the exception of *TRI*_2_ and *TRI*_3_ whilst the *GRQ* values vary uniformly around the 0.80 value excepting *TRI*_4_. The *PEQ* and *SPQ* values oscillate among the [0.5,0.8] interval.The highest value of *TRIQ* is reached by *TRI*_4_, thus it is the best solution for this experiment.

**Table 12:** *OPT* GDS4510 solution table

SOLUTION	TRIQ	BIOQ	GRQ	PEQ	SPQ
*T* *R* *I*_1_	0.42009257	0.00134246	0.83749772	0.8574642	0.8309809
*T* *R* *I*_2_	0.42005895	0.00356033	0.85404927	0.82019206	0.71298946
*T* *R* *I*_3_	0.42901805	0.00256279	0.87133294	0.85498273	0.72908679
*T* *R* *I*_4_	0.44490172	0.00136003	0.93478154	0.77222529	0.63395639
*T* *R* *I*_5_	0.37648925	0.00128696	0.81223451	0.51226675	0.50677264
*T* *R* *I*_6_	0.37500834	0.00122195	0.80916885	0.50865504	0.50594149
*T* *R* *I*_7_	0.37783613	0.00125442	0.81768005	0.50153608	0.50120198
*T* *R* *I*_8_	0.37545313	0.00144891	0.80990327	0.50841987	0.50692736
*T* *R* *I*_9_	0.43860855	0.00167827	0.89471647	0.86719834	0.73045828
*T* *R* *I*_10_	0.37115418	0.00120689	0.80002563	0.50727999	0.50352975

#### GDS4510 summary

**Fig. 7:**
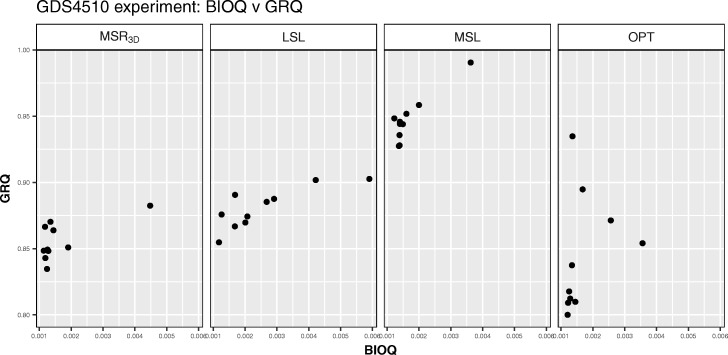
*BIOQ* vs *GRQ* dispersion graph for each GDS4510 solution of each experiment

**Fig. 8:**
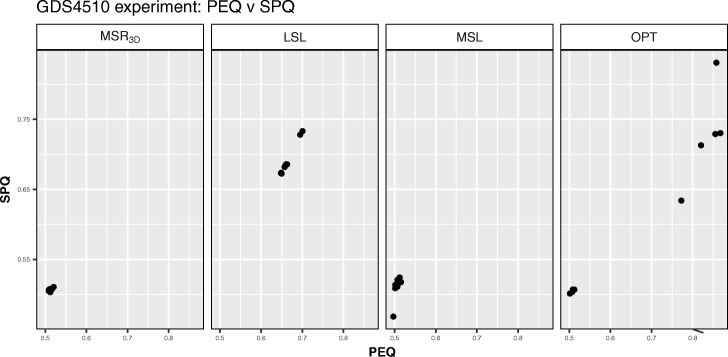
*PEQ* vs *SPQ* dispersion graph for each GDS4510 solution of each experiment

We can see how the solutions are distributed regarding *BIOQ* and *GRQ* in Fig. 7; we observe that all points of all experiments are concentrated in a *BIOQ* interval of [0.0011,0.0059]. Regarding the *GRQ* values, the *MSR*_3*D*_ and *LSL* experiments have all the solutions in the [0.83,0.90] interval, the *MSL* experiment has all the solutions in the [0.92,0.99] interval and, the *OPT* experiment has all the solutions in the [0.80,0.95] interval. Regarding the *PEQ* and *SPQ* distribution we can see in Fig. 8 how the majority of solutions are concentrated around the point *PEQ*=0.5,*SPQ*=0.5 in the *MSR*_3*D*_ and *MSL* experiments, meanwhile the solutions of *LSL* experiment are concentrated in the interval [0.625,0.75] for *PEQ* and *SPQ* values and, the *OPT* experiment has his solutions dispersed in two groups: one group around the *PEQ*=0.5,*SPQ*=0.5 point and the other in an interval of [0.60,0.83] for both *PEQ* and *SPQ* values.

A global *TRIQ*-based ranking of solutions is shown in Table 13. The *MSL*, *LSL* and a part of *OPT* solutions are placed alternatively on the first positions and the *MSR*_3*D*_ and the remaining of *OPT* solutions are in the last positions.

**Table 13:** GDS4510 ranking table

EXPERIMENT	SOLUTION	TRIQ	BIOQ	GRQ	PEQ	SPQ
*MSL*	*T* *R* *I*_1_	0.446289279	0.003624207	0.990551544	0.496833632	0.468297522
*OPT*	*T* *R* *I*_4_	0.44490172	0.00136003	0.93478154	0.77222529	0.63395639
*OPT*	*T* *R* *I*_9_	0.43860855	0.00167827	0.89471647	0.86719834	0.73045828
*MSL*	*T* *R* *I*_8_	0.436192976	0.001999141	0.958438573	0.512285251	0.524074273
*LSL*	*T* *R* *I*_1_	0.435171938	0.005902591	0.902662935	0.6949723	0.728137064
*MSL*	*T* *R* *I*_9_	0.433173322	0.001604555	0.95182792	0.510885656	0.521911883
*MSL*	*T* *R* *I*_5_	0.431185402	0.001224915	0.948344121	0.507510722	0.517195194
*MSL*	*T* *R* *I*_2_	0.430638622	0.001399471	0.945717127	0.515568434	0.51747227
*LSL*	*T* *R* *I*_4_	0.42987221	0.004218243	0.901813346	0.657798589	0.68295641
*MSL*	*T* *R* *I*_3_	0.429698209	0.00149303	0.943951098	0.506740977	0.520684131
*MSL*	*T* *R* *I*_7_	0.429129078	0.001401202	0.944156645	0.501640545	0.513675839
*OPT*	*T* *R* *I*_3_	0.42901805	0.00256279	0.87133294	0.85498273	0.72908679
*LSL*	*T* *R* *I*_2_	0.427168871	0.002676716	0.885320349	0.700788434	0.733259027
*MSL*	*T* *R* *I*_4_	0.425844616	0.001388422	0.935696236	0.506485147	0.510953062
*LSL*	*T* *R* *I*_9_	0.42454573	0.001683951	0.890635412	0.663209255	0.68578253
*MSL*	*T* *R* *I*_6_	0.422692807	0.001367523	0.927464145	0.507112049	0.513355693
*LSL*	*T* *R* *I*_3_	0.422560787	0.002909652	0.887599773	0.648196371	0.673124663
*MSL*	*T* *R* *I*_10_	0.422409162	0.001390319	0.928018397	0.501351072	0.508781791
*OPT*	*T* *R* *I*_1_	0.42009257	0.00134246	0.83749772	0.8574642	0.8309809
*OPT*	*T* *R* *I*_2_	0.42005895	0.00356033	0.85404927	0.82019206	0.71298946
*LSL*	*T* *R* *I*_10_	0.417718487	0.002071672	0.874308872	0.657210052	0.681971994
*LSL*	*T* *R* *I*_7_	0.417068507	0.001262531	0.87574877	0.648950843	0.673803828
*LSL*	*T* *R* *I*_5_	0.416008121	0.002006917	0.869706286	0.658784335	0.683658641
*LSL*	*T* *R* *I*_6_	0.41490654	0.001678815	0.866767983	0.661174595	0.686024185
*LSL*	*T* *R* *I*_8_	0.40861261	0.001179271	0.854772399	0.649739272	0.672541022
*M* *S* *R*_3 *D*_	*T* *R* *I*_7_	0.40677296	0.004479209	0.882477468	0.520330064	0.510517317
*M* *S* *R*_3 *D*_	*T* *R* *I*_1_	0.399937853	0.001348086	0.870211819	0.516112583	0.507469069
*M* *S* *R*_3 *D*_	*T* *R* *I*_2_	0.397972383	0.001177971	0.866535835	0.511042941	0.504338338
*M* *S* *R*_3 *D*_	*T* *R* *I*_4_	0.397028323	0.0014405	0.863853884	0.512208068	0.503122322
*M* *S* *R*_3 *D*_	*T* *R* *I*_6_	0.392316477	0.00190466	0.850869722	0.513791033	0.506534134
*M* *S* *R*_3 *D*_	*T* *R* *I*_3_	0.391066411	0.001255371	0.849235889	0.508613518	0.506273874
*M* *S* *R*_3 *D*_	*T* *R* *I*_9_	0.390891083	0.001281294	0.848296937	0.510926324	0.507706903
*M* *S* *R*_3 *D*_	*T* *R* *I*_10_	0.390730352	0.001137925	0.8484396	0.50930819	0.506402803
*M* *S* *R*_3 *D*_	*T* *R* *I*_5_	0.388644055	0.001187588	0.842929885	0.511734835	0.505831309
*M* *S* *R*_3 *D*_	*T* *R* *I*_8_	0.3851186	0.001240227	0.834606686	0.508323861	0.504792392
*OPT*	*T* *R* *I*_7_	0.37783613	0.00125442	0.81768005	0.50153608	0.50120198
*OPT*	*T* *R* *I*_5_	0.37648925	0.00128696	0.81223451	0.51226675	0.50677264
*OPT*	*T* *R* *I*_8_	0.37545313	0.00144891	0.80990327	0.50841987	0.50692736
*OPT*	*T* *R* *I*_6_	0.37500834	0.00122195	0.80916885	0.50865504	0.50594149
*OPT*	*T* *R* *I*_10_	0.37115418	0.00120689	0.80002563	0.50727999	0.50352975

**Fig. 9:**
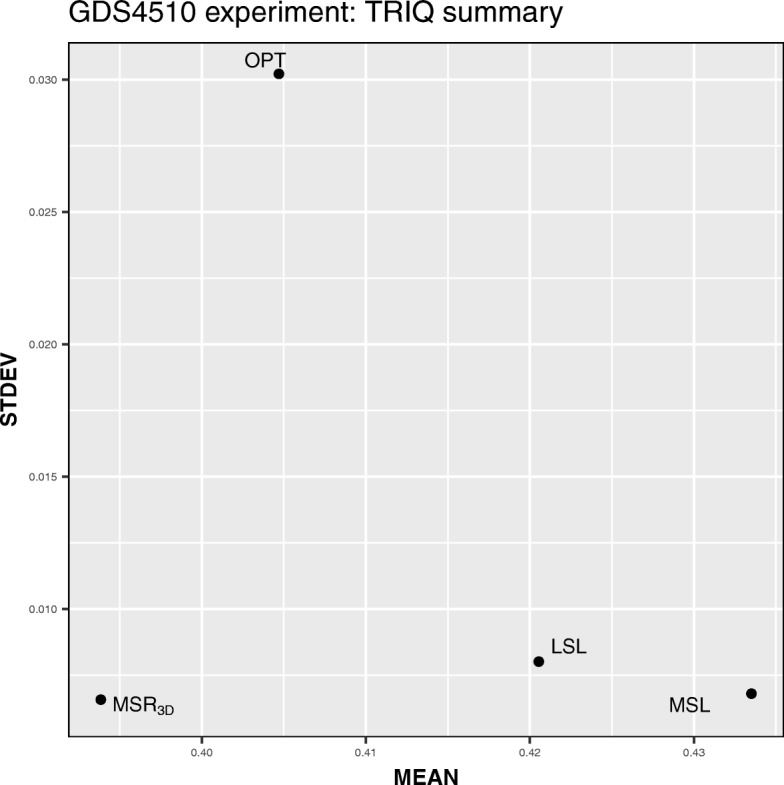
*MEAN* vs *STDEV* dispersion graph for each GDS4510 experiment

We can see in Table 14 how the GDS4510 *MSL* experiment has the best value of the mean of *TRIQ* and the four experiments have low values of standard deviation having the *MSR*_3*D*_ experiment the lowest value but very close to the *MSL* one. This fact implies that the four experiments have a low sparse distribution and solutions with high quality. We can see in Fig. 9 how the *MSR*_3*D*_, *LSL* and, *MSL* points are located on the bottom side of the graph meanwhile the *OPT* point is located in a high level of the standard deviation axis; on the other hand, *LSL* and, *MSL* points are located on the right side of the graph meanwhile the *MSR*_3*D*_ and *OPT* points are located in a left level of the average axis. Hence, in terms of standard deviation and average, we can conclude that *MSL* is the best experiment.

**Table 14:** GDS4510 summary table

EXPERIMENT	BEST SOLUTION	BEST TRIQ	MEAN	STDEV
*M* *S* *R*_3 *D*_	*T* *R* *I*_7_	0.40677296	0.39404785	0.006348192
*LSL*	*T* *R* *I*_1_	0.435171938	0.42136338	0.007979308
*MSL*	*T* *R* *I*_1_	0.446289279	0.430725347	0.006987671
*OPT*	*T* *R* *I*_4_	0.44490172	0.402862087	0.030140772

**Fig. 10:**
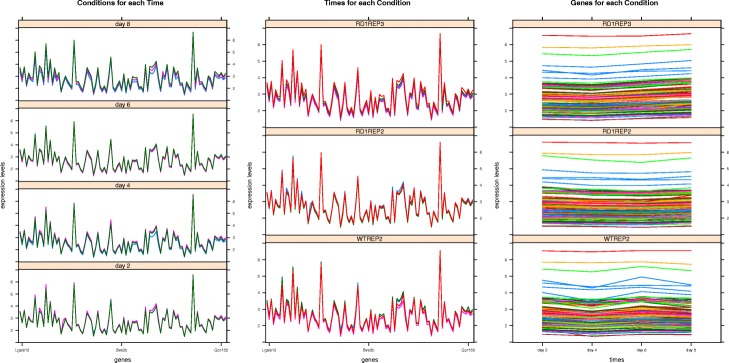
*TRI*_1_ graphic views of the GDS4510 *MSL* experiment

The most valuable solution of all experiments is the tricluster *TRI*_1_ of the *MSL* experiment. We can see in Fig. 10 how this solution depicts very uniform patterns consistent with the *GRQ* value. Also, we can see in Table 15 that this solution has Gene Ontology terms with low *p*-value such as *sensory perception of chemical stimulus*, *olfactory receptor activity* or *detection of chemical stimulus involved in sensory perception of smell*. The term *olfactory receptor activity* is a molecular function that combining with an odorant and transmitting the signal from one side of the membrane to the other to initiate a change in cell activity in response to detection of smell; this function is part of the biological process *detection of chemical stimulus involved in sensory perception of smell* that is the series of events involved in the perception of smell in which an olfactory chemical stimulus is received and converted into a molecular signal. Finally, that process is framed in a more general biological process called *sensory perception of chemical stimulus* that is the series of events required for an organism to receive a sensory chemical stimulus, convert it to a molecular signal, and recognize and characterize the signal.

**Table 15:** *TRI*_1_ GO table of the *MSL* GDS4510 experiment

TERM ID	TERM	*P*-VALUE
GO:0007606	Sensory perception of chemical stimulus	1.68E-25
GO:0004984	Olfactory receptor activity	6.56E-19
GO:0050911	Detection of chemical stimulus involved in sensory perception of smell	6.56E-19
GO:0050907	Detection of chemical stimulus involved in sensory perception	2.97E-18
GO:0004930	G-protein coupled receptor activity	4.68E-17
GO:0007186	G-protein coupled receptor signaling pathway	4.68E-17
GO:0007608	Sensory perception of smell	6.93E-16
GO:0009593	Detection of chemical stimulus	1.16E-15
GO:0007600	Sensory perception	5.28E-15
GO:0050906	Detection of stimulus involved in sensory perception	8.32E-14
GO:0004872	Receptor activity	9.34E-14
GO:0060089	Molecular transducer activity	6.27E-13
GO:0004888	Transmembrane signaling receptor activity	8.08E-13
GO:0050877	Nervous system process	1.07E-12
GO:0099600	Transmembrane receptor activity	2.01E-12
GO:0038023	Signaling receptor activity	1.43E-11
GO:0004871	Signal transducer activity	2.69E-11
GO:0051606	Detection of stimulus	1.64E-10
GO:0003008	System process	1.09E-09
GO:0005549	Odorant binding	1.85E-08

### Human GDS4472 dataset

The dataset, corresponding to this batch, has been obtained from GEO [35] under code GDS4472 titled *Transcription factor oncogene OTX2 silencing effect on D425 medulloblastoma cell line: time course* [34]. In this experiment, the effect of doxycycline on medulloblastoma cancerous cells at six times after induction (0, 8, 16, 24, 48 and 96 h) had been studied.

Our input dataset $D_{GSD4472_{3D}}$ is composed of 54675 genes, 4 conditions (one for each individual involved) and 6 time points (one per hour) and has been used as the input of the *TriGen* and the *OPTtricluster* algorithm in four experiments: *MSR*_3*D*_, *LSL*, *MSL* and, *OPT*.

#### GDS4472 *MSR*_3*D*_ experiment

For this experiment, *TRI*_4_ has the best value of *BIOQ*, *TRI*_6_ has the best value of *PEQ*, *TRI*_3_ has the best value of *SPQ* and *TRI*_5_ has the best value of *GRQ* as you can see Table 16. The *PEQ* and *SPQ* values of the solutions oscillate around 0.64 and the *GRQ* values vary between 0.76 and 0.64; the *BIOQ* index oscillates around 0.0014 reaching two peaks at *TRI*_4_ and *TRI*_8_. In general, the *TRIQ* value of solutions are in [0.32,0.37] having *TRI*_3_ and *TRI*_7_ as outstanding ones and *TRI*_5_ as the best solution in this experiment.

**Table 16:** *MSR*_3*D*_ GDS4472 solution table

SOLUTION	TRIQ	BIOQ	GRQ	PEQ	SPQ
*T* *R* *I*_1_	0.339109333	0.001444791	0.696219908	0.596698979	0.601280513
*T* *R* *I*_2_	0.321761941	0.001591523	0.645157719	0.633303534	0.624758294
*T* *R* *I*_3_	0.363970471	0.001440455	0.742093089	0.650828401	0.677431755
*T* *R* *I*_4_	0.343765956	0.001732664	0.69802844	0.623399523	0.650365438
*T* *R* *I*_5_	0.370128492	0.001337649	0.761586904	0.637388072	0.659110049
*T* *R* *I*_6_	0.360725206	0.001406179	0.730735981	0.688724917	0.665829566
*T* *R* *I*_7_	0.366252916	0.001263468	0.750692098	0.655100071	0.651786783
*T* *R* *I*_8_	0.351001074	0.00159526	0.709109493	0.674238924	0.656954002
*T* *R* *I*_9_	0.327754495	0.001401494	0.664697595	0.606214508	0.617279679
*T* *R* *I*_10_	0.360821995	0.001434449	0.743345617	0.631027541	0.624302919

#### GDS4472 *LSL* experiment

We can verify in Table 17 how *TRI*_1_ has the best values of *BIOQ*, *GRQ*, *PEQ* and *SPQ*. In general, the *GRQ*, *PEQ* and *SPQ* indexes of the solutions depicts homogeneous values with the exception of *TRI*_1_ where they reach their maximum; regarding *BIOQ* values, those reach three peaks at *TRI*_1_, *TRI*_4_ and *TRI*_10_. The *TRIQ* values vary between 0.39 and 0.44 being *TRI*_1_ the best solution of this experiment.

**Table 17:** *LSL* GDS4472 solution table

SOLUTION	TRIQ	BIOQ	GRQ	PEQ	SPQ
*T* *R* *I*_1_	0.447346181	0.027287612	0.923852614	0.69377633	0.589450252
*T* *R* *I*_2_	0.392576223	0.004031468	0.862302229	0.468881448	0.443910489
*T* *R* *I*_3_	0.409737004	0.002294049	0.886097803	0.570674741	0.512342415
*T* *R* *I*_4_	0.421749212	0.00967779	0.895313993	0.60768017	0.568014215
*T* *R* *I*_5_	0.402016193	0.002568856	0.869691073	0.55912722	0.497979503
*T* *R* *I*_6_	0.394065329	0.00467901	0.864596579	0.474398477	0.443345363
*T* *R* *I*_7_	0.39497655	0.005644179	0.865341762	0.477395239	0.442959875
*T* *R* *I*_8_	0.397055929	0.005748916	0.868777413	0.482543181	0.450866935
*T* *R* *I*_9_	0.40510461	0.007434596	0.881069048	0.514114336	0.465079524
*T* *R* *I*_10_	0.411954946	0.019662416	0.89416486	0.458207225	0.430948663

#### GDS4472 *MSL* experiment

In Table 18 we can see how *TRI*_9_ has the best values of *BIOQ* and *GRQ* while *TRI*_7_ has the best value of *PEQ* and *TRI*_10_ has the best value of *SPQ*. The *PEQ* values of the solutions vary in the [0.43,0.46] interval and the *SEQ* values are in the [0.40,0.44] interval while all solutions have high *GRQ* values close to 0.90; the *BIOQ* values have three peaks at *TRI*_5_, *TRI*_7_ and *TRI*_9_. Regarding *TRIQ* values, they vary in [0.40,0.42] interval being *TRI*_1_, *TRI*_5_ and *TRI*_7_ the outstanding solutions and being *TRI*_9_ the best solution.

**Table 18:** *MSL* GDS4472 solution table

SOLUTION	TRIQ	BIOQ	GRQ	PEQ	SPQ
*T* *R* *I*_1_	0.413005918	0.008623332	0.909739803	0.463874665	0.432091958
*T* *R* *I*_2_	0.406682712	0.005351847	0.901242812	0.449739986	0.420453301
*T* *R* *I*_3_	0.404078935	0.004069221	0.896447319	0.445616204	0.423691724
*T* *R* *I*_4_	0.409123273	0.004869646	0.9053715	0.456215467	0.43458153
*T* *R* *I*_5_	0.410786658	0.011209144	0.903088937	0.453954127	0.424976095
*T* *R* *I*_6_	0.404207143	0.004999521	0.896798986	0.44398627	0.415769491
*T* *R* *I*_7_	0.411937377	0.012628523	0.901459314	0.468134175	0.432653616
*T* *R* *I*_8_	0.405644251	0.0030952	0.902252364	0.445061054	0.418853066
*T* *R* *I*_9_	0.42006885	0.025664213	0.912118818	0.439476488	0.408307841
*T* *R* *I*_10_	0.41078403	0.006450477	0.90556104	0.465916366	0.440771152

#### GDS4472 *OPT* experiment

For this experiment, *TRI*_5_ has the best value of *BIOQ*, *TRI*_10_ has the best value of *GRQ* and *SPQ* and, *TRI*_8_ has the best value of *PEQ* as you can see Table 19. The *BIOQ* index oscillates around 0.0015 reaching three peaks at *TRI*_5_, *TRI*_9_ and, *TRI*_10_. The *GRQ* index vary in the [0.6,07] interval reaching an outstanding value in the *TRI*_10_ solution. Regarding the *PEQ* values they vary in a interval of [0.42,0.86] and the *SPQ* values in the [0.34,0.76] interval. The *TRIQ* values vary between 0.28 and 0.44 being *TRI*_10_ the best solution of this experiment.

**Table 19:** *OPT* GDS4472 solution table

SOLUTION	TRIQ	BIOQ	GRQ	PEQ	SPQ
*T* *R* *I*_1_	0.361091084	0.001165855	0.728642443	0.841096481	0.539927104
*T* *R* *I*_2_	0.302530473	0.001445096	0.649190316	0.42227834	0.420357627
*T* *R* *I*_3_	0.298417139	0.001567996	0.639083858	0.421848063	0.418143898
*T* *R* *I*_4_	0.290997577	0.0013925	0.620125263	0.423388521	0.421635907
*T* *R* *I*_5_	0.353327655	0.00233497	0.7175687	0.832938328	0.469715461
*T* *R* *I*_6_	0.298612766	0.001430159	0.640127397	0.421427176	0.415507376
*T* *R* *I*_7_	0.282392223	0.0018726	0.610369316	0.397933947	0.348229987
*T* *R* *I*_8_	0.35196608	0.00159536	0.707996999	0.865220464	0.49417155
*T* *R* *I*_9_	0.328919371	0.001916523	0.649746035	0.838138155	0.523115758
*T* *R* *I*_10_	0.446233789	0.002289266	0.924944835	0.740548556	0.761675883

#### GDS4472 summary

**Fig. 11:**
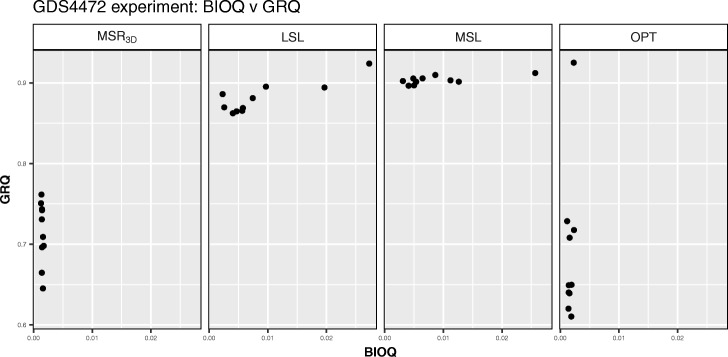
*BIOQ* vs *GRQ* dispersion graph for each GDS4472 solution of each experiment

**Fig. 12:**
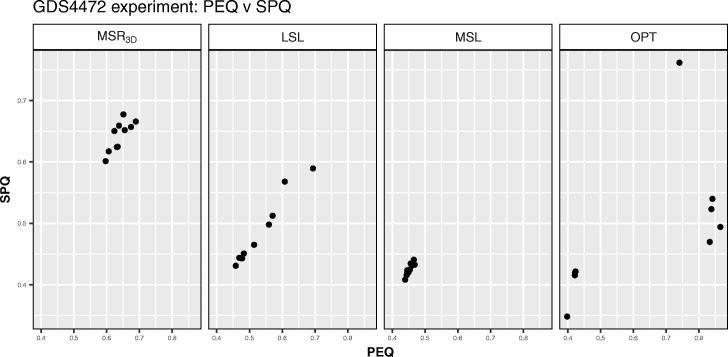
*PEQ* vs *SPQ* dispersion graph for each GDS4472 solution of each experiment

We can observe in Fig. 11 how the solutions of the four experiments are in a *BIOQ* interval of [0.0012,0.0272] meanwhile the *GRQ* values of the solutions of *MSR*_3*D*_ are in the [0.6451,0.7615] interval, the solutions of *LSL* are in the [0.8623,0.8953] interval, the solutions of *MSL* are in the [0.8964,0.9238] interval and, the solutions of *OPT* are in the [0.6,0.7] interval with an outstanding point near to *GRQ*=0.92. Regarding the *PEQ* and *SPQ* solutions distribution we can see in Fig. 12 how the *PEQ* and *SPQ* of *MSR*_3*D*_ are concentrated in the [0.50,0.75] interval, the values *PEQ* and *SPQ* of *LSL* are in the [0.325,0.75] interval, the values *PEQ* and *SPQ* of *MSL* are in the [0.325,0.50] interval and, the values *PEQ* and *SPQ* of *OPT* are dispersed in three groups: the first in the [0.42,0.45] interval for *PEQ* and *SPQ*, the second in the [0.70,0.85] interval for *PEQ* and the [0.46,0.54] interval for *SPQ* and the third, that is a single point, in *PEQ*=0.74,*SPQ*=0.76.

We can see the global *TRIQ*-based ranking of solutions in Table 20; the *MSL* solutions, one *OPT* solution and, the *LSL* solutions are placed alternatively on the first positions and the *MSR*_3*D*_ and the remaining of *OPT* solutions are on the last positions.

**Table 20:** GDS4472 ranking table

EXPERIMENT	SOLUTION	TRIQ	BIOQ	GRQ	PEQ	SPQ
*LSL*	*T* *R* *I*_1_	0.447346181	0.027287612	0.923852614	0.69377633	0.589450252
*OPT*	*T* *R* *I*_10_	0.446233789	0.002289266	0.924944835	0.740548556	0.761675883
*LSL*	*T* *R* *I*_4_	0.421749212	0.00967779	0.895313993	0.60768017	0.568014215
*MSL*	*T* *R* *I*_9_	0.42006885	0.025664213	0.912118818	0.439476488	0.408307841
*MSL*	*T* *R* *I*_1_	0.413005918	0.008623332	0.909739803	0.463874665	0.432091958
*LSL*	*T* *R* *I*_10_	0.411954946	0.019662416	0.89416486	0.458207225	0.430948663
*MSL*	*T* *R* *I*_7_	0.411937377	0.012628523	0.901459314	0.468134175	0.432653616
*MSL*	*T* *R* *I*_5_	0.410786658	0.011209144	0.903088937	0.453954127	0.424976095
*MSL*	*T* *R* *I*_10_	0.41078403	0.006450477	0.90556104	0.465916366	0.440771152
*LSL*	*T* *R* *I*_3_	0.409737004	0.002294049	0.886097803	0.570674741	0.512342415
*MSL*	*T* *R* *I*_4_	0.409123273	0.004869646	0.9053715	0.456215467	0.43458153
*MSL*	*T* *R* *I*_2_	0.406682712	0.005351847	0.901242812	0.449739986	0.420453301
*MSL*	*T* *R* *I*_8_	0.405644251	0.0030952	0.902252364	0.445061054	0.418853066
*LSL*	*T* *R* *I*_9_	0.40510461	0.007434596	0.881069048	0.514114336	0.465079524
*MSL*	*T* *R* *I*_6_	0.404207143	0.004999521	0.896798986	0.44398627	0.415769491
*MSL*	*T* *R* *I*_3_	0.404078935	0.004069221	0.896447319	0.445616204	0.423691724
*LSL*	*T* *R* *I*_5_	0.402016193	0.002568856	0.869691073	0.55912722	0.497979503
*LSL*	*T* *R* *I*_8_	0.397055929	0.005748916	0.868777413	0.482543181	0.450866935
*LSL*	*T* *R* *I*_7_	0.39497655	0.005644179	0.865341762	0.477395239	0.442959875
*LSL*	*T* *R* *I*_6_	0.394065329	0.00467901	0.864596579	0.474398477	0.443345363
*LSL*	*T* *R* *I*_2_	0.392576223	0.004031468	0.862302229	0.468881448	0.443910489
*M* *S* *R*_3 *D*_	*T* *R* *I*_5_	0.370128492	0.001337649	0.761586904	0.637388072	0.659110049
*M* *S* *R*_3 *D*_	*T* *R* *I*_7_	0.366252916	0.001263468	0.750692098	0.655100071	0.651786783
*M* *S* *R*_3 *D*_	*T* *R* *I*_3_	0.363970471	0.001440455	0.742093089	0.650828401	0.677431755
*OPT*	*T* *R* *I*_1_	0.361091084	0.001165855	0.728642443	0.841096481	0.539927104
*M* *S* *R*_3 *D*_	*T* *R* *I*_10_	0.360821995	0.001434449	0.743345617	0.631027541	0.624302919
*M* *S* *R*_3 *D*_	*T* *R* *I*_6_	0.360725206	0.001406179	0.730735981	0.688724917	0.665829566
*OPT*	*T* *R* *I*_5_	0.353327655	0.00233497	0.7175687	0.832938328	0.469715461
*OPT*	*T* *R* *I*_8_	0.35196608	0.00159536	0.707996999	0.865220464	0.49417155
*M* *S* *R*_3 *D*_	*T* *R* *I*_8_	0.351001074	0.00159526	0.709109493	0.674238924	0.656954002
*M* *S* *R*_3 *D*_	*T* *R* *I*_4_	0.343765956	0.001732664	0.69802844	0.623399523	0.650365438
*M* *S* *R*_3 *D*_	*T* *R* *I*_1_	0.339109333	0.001444791	0.696219908	0.596698979	0.601280513
*OPT*	*T* *R* *I*_9_	0.328919371	0.001916523	0.649746035	0.838138155	0.523115758
*M* *S* *R*_3 *D*_	*T* *R* *I*_9_	0.327754495	0.001401494	0.664697595	0.606214508	0.617279679
*M* *S* *R*_3 *D*_	*T* *R* *I*_2_	0.321761941	0.001591523	0.645157719	0.633303534	0.624758294
*OPT*	*T* *R* *I*_2_	0.302530473	0.001445096	0.649190316	0.42227834	0.420357627
*OPT*	*T* *R* *I*_6_	0.298612766	0.001430159	0.640127397	0.421427176	0.415507376
*OPT*	*T* *R* *I*_3_	0.298417139	0.001567996	0.639083858	0.421848063	0.418143898
*OPT*	*T* *R* *I*_4_	0.290997577	0.0013925	0.620125263	0.423388521	0.421635907
*OPT*	*T* *R* *I*_7_	0.282392223	0.0018726	0.610369316	0.397933947	0.348229987

**Fig. 13:**
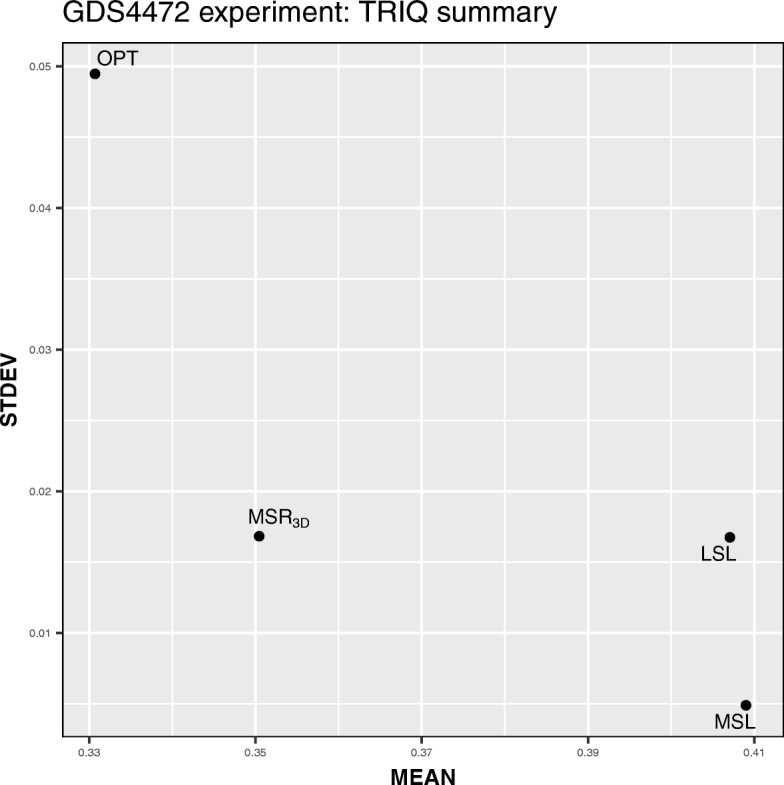
*MEAN* vs *STDEV* dispersion graph for each GDS4472 experiment

We can see in Table 21 how the *MSL* experiment has the best value of the average and standard deviation of *TRIQ*, however, the *LSL* experiment has the best tricluster closely followed by the *OPT* experiment. In Fig. 13 we can see how the *MSL* is placed in the bottom-right position being the best experiment in terms of standard deviation and average.

**Table 21:** GDS4472 summary table

EXPERIMENT	BEST SOLUTION	BEST TRIQ	MEAN	STDEV
*M* *S* *R*_3 *D*_	*T* *R* *I*_5_	0.370128492	0.350529188	0.016814529
*LSL*	*T* *R* *I*_1_	0.447346181	0.407658218	0.016734175
*MSL*	*T* *R* *I*_9_	0.42006885	0.409631915	0.004869533
*OPT*	*T* *R* *I*_10_	0.446233789	0.331448816	0.049451114

**Fig. 14:**
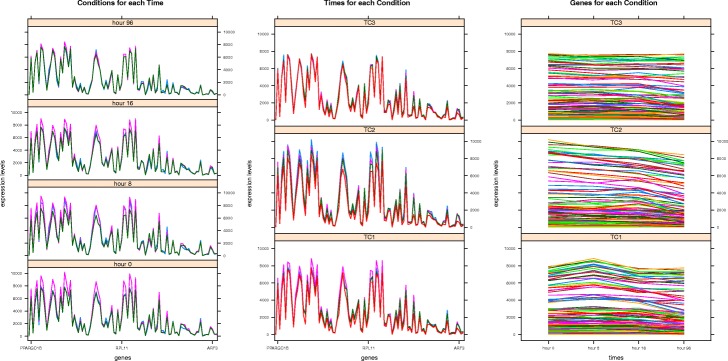
*TRI*_1_ graphic views of the GDS4472 *LSL* experiment

The most valuable solution of all experiments is the tricluster *TRI*_1_ of the *LSL* experiment. This solution depicts very uniform patterns since has a very high *GRQ* value, we can check this fact in Fig. 14. Also, we can see in Table 22 that this solution has Gene Ontology terms with very low *p*-value such as *SRP-dependent cotranslational protein targeting to membrane*, *nuclear-transcribed mRNA catabolic process, nonsense-mediated decay* or *ribonucleoprotein complex*.

**Table 22:** *TRI*_1_ GO table of the *LSL* GDS4472 experiment

TERM ID	TERM	*P*-VALUE
GO:1990904	Ribonucleoprotein complex	1.52E-41
GO:0030529	Intracellular ribonucleoprotein complex	1.52E-41
GO:0044403	Symbiosis, encompassing mutualism through parasitism	6.40E-40
GO:0044419	Interspecies interaction between organisms	1.52E-39
GO:0016032	Viral process	2.20E-39
GO:0045047	Protein targeting to ER	2.91E-38
GO:0006613	Cotranslational protein targeting to membrane	3.78E-38
GO:0072599	Establishment of protein localization to endoplasmic reticulum	8.16E-38
GO:0006614	SRP-dependent cotranslational protein targeting to membrane	8.33E-37
GO:0070972	Protein localization to endoplasmic reticulum	6.26E-36
GO:0005840	Ribosome	6.47E-36
GO:0022626	Cytosolic ribosome	2.56E-35
GO:0019080	Viral gene expression	1.03E-34
GO:0043624	Cellular protein complex disassembly	1.10E-34
GO:0022618	Ribonucleoprotein complex assembly	1.54E-34
GO:0071826	Ribonucleoprotein complex subunit organization	1.96E-34
GO:0000184	Nuclear-transcribed mRNA catabolic process, nonsense-mediated decay	4.39E-34
GO:0044391	Ribosomal subunit	5.80E-34
GO:0001677	Formation of translation initiation ternary complex	6.45E-34
GO:0006412	Translation	6.45E-34

The *SRP-dependent cotranslational protein targeting to membrane* process is described as the targeting of proteins to a membrane that occurs during translation and is dependent upon two key components, the signal-recognition particle (SRP) and the SRP receptor. SRP is a cytosolic particle that transiently binds to the endoplasmic reticulum (ER) signal sequence in a nascent protein, to the large ribosomal unit, and to the SRP receptor in the ER membrane; it is a protein targeting process that occurs in the intracellular component and is part of the cellular protein localization process. The *nuclear-transcribed mRNA catabolic process, nonsense-mediated decay* is a biological process that describes the nonsense-mediated decay pathway for nuclear-transcribed mRNAs degrades mRNAs in which an amino-acid codon has changed to a nonsense codon; this prevents the translation of such mRNAs into truncated, and potentially harmful, proteins; it is a negative regulation of gene expression process that negatively regulates the macromolecule metabolic process. Finally the *ribonucleoprotein complex* is a cellular component that is defined as a macromolecular complex containing both protein and RNA molecules.

## Conclusions and discussion

Although triclustering has emerged as an essential task to study 3D datasets, there is no consensus on how to evaluate tricluster solutions obtained from each data set. Different authors validate their triclusters on different measures, with correlation, graphic validation and Gene Ontology terms being the most common ones. In this work we have presented a tricluster validation measure, *TRIQ*, a single evaluation measure that combines the information from the three aforementioned sources of validation.

We have applied *TRIQ* to three different datasets: the yeast cell cycle (*Saccharomyces Cerevisiae*), in particular the elutriation experiment, an experiment with mice (*Mus Musculus*) called GDS4510 and data from an experiments with humans (*Homo Sapiens*) called GDS4472.

We have shown that *TRIQ* has successfully resumed the three validation measures (correlation, graphic validation and Gene Ontology terms) yielding the same validation results as in [27] where each of the components of *TRIQ* (*BIOQ*, *GRQ*, *PEQ*, and *SPQ*) where applied separately. In that publication we presented the *MSL* measure, comparing it to *MSR*_3*D*_ and *LSL*, with the same datasets used in this article. We concluded that *MSL* was the best fitness function. In this publication, we have seen how *MSL* has obtained the best general results, with high values of *TRIQ* and low standard deviation for all solutions presented. Therefore, we can conclude that *TRIQ* has been successful in representing and summarizing the individual values provided by *BIOQ*, *GRQ*, *PEQ*, and *SPQ*. Furthermore, we have applied *TRIQ* to results from another algorithm, *OPTRicluster*, and we have shown how TRIQ has been a valid tool to compare results from different algorithms in a quantitative straightforward manner.

For the case of triclustering being applied to not biologically related fields as in [36], *TRIQ* can also cope with the analysis of the tricluster solutions thanks to the weighting system (see “Methods” section), which allows for each term to be included or removed in the final measure.
